# Coronary Artery Ectasia: An Insight into Intraprocedural and Postprocedural Management Strategies

**DOI:** 10.7759/cureus.3928

**Published:** 2019-01-21

**Authors:** Muhammad Waqas, Lilian L Bizzocchi, Mark A Menegus, Robert T Faillace

**Affiliations:** 1 Internal Medicine, Albert Einstein College of Medicine / Jacobi Medical Center, Bronx, USA; 2 Cardiology, Albert Einstein College of Medicine / Jacobi Medical Center, Bronx, USA

**Keywords:** coronary artery ectasia, thrombolysis, acute coronary syndrome, thrombectomy, time in therapeutic range, infarct-related arteries

## Abstract

Coronary artery ectasia (CAE) can present as an acute coronary syndrome (ACS) with a high clot burden in ectatic coronary arteries. Thrombectomy with intracoronary thrombolysis often does not ensure immediate blood flow. Also, there have not been clear guidelines regarding long-term management in such cases.

A 40-year-old male presented with anginal chest discomfort and a working diagnosis of non-ST elevation myocardial infarction (NSTEMI) was made. The initial angiography showed thrombotic occlusion of several large and ectatic coronary arteries with visibly swirling blood flow. The culprit lesions were treated with balloon angioplasty and multiple rounds of thrombectomy yielding red thrombi. Interestingly, the post-intervention antegrade flow decreased in both vessels (Thrombolysis in Myocardial Infarction (TIMI) score: 0), possibly because of the distal migration of the clots. Peri-procedure, the patient received two boluses of eptifibatide, 180 mcg/kg each, followed by a continuous infusion of 2 mcg/kg/minute for 18 hours. Afterward, the patient was started on ticagrelor and continued on daily aspirin, high-intensity statin, beta blocker, and Coumadin® with heparin bridge. During the one year follow-up period, the Coumadin was switched to rivaroxaban, ticagrelor was stopped after six months, and the patient was continued on guideline-directed medical therapy (GDMT) for coronary artery disease (CAD) with favorable outcomes.

The presented case gives us an insight into not only the intra-procedural but also the post-procedural management of ACS in the setting of CAE, and that is thrombectomy alone followed by longer duration oral anticoagulation in addition to GDMT for CAD. However, it will be interesting to see future studies aimed toward defining the duration as well as the choice of anticoagulation, i.e., dual antiplatelet therapy (DAPT) alone or in combination with warfarin/novel oral anticoagulants (NOACs).

## Introduction

Coronary artery ectasia (CAE) often presents in the form of an acute coronary syndrome (ACS) due to slow flow leading to thrombus formation in ectatic coronary arteries, and getting rid of this high thrombus burden during the percutaneous coronary intervention (PCI) can be a challenging task. For this purpose, thrombectomy with intracoronary thrombolysis has been utilized for restoring the blood flow. However, there have been cases showing migration of the clots into distal coronary vessels during thrombectomy attempts, making it difficult to establish immediate flow in all segments. Such cases were managed with extended duration oral anticoagulation, and thrombus clearance was demonstrated with serial follow-up angiographies. We hereby describe a case of diffuse CAE presented as ACS. The high clot burden was successfully dealt with using thrombectomy and a glycoprotein IIb/IIIa inhibitor followed by extended duration oral anticoagulation, thus avoiding intracoronary thrombolysis and negating the need for follow-up angiographies.

## Case presentation

A 40-year-old male with a past medical history of hypertension and a family history of premature myocardial infarctions (MIs) in a number of first-degree relatives came to the emergency department (ED) with chest pain of two hours’ duration. The patient described it as sudden onset retrosternal pressure which was constant, non-progressive, 10/10, non-radiating, and without any aggravating or alleviating factors. Symptoms started at rest and were associated with mild shortness of breath, left arm heaviness, vomiting, and a syncopal episode. The patient reported that his mother experienced myocardial infarction at 38 years of age and two of his maternal uncles and three first cousins died of myocardial infarction in their 40s. Enroute to the ED, the patient received aspirin (162 mg) and sublingual nitroglycerin with minimal improvement. Vital signs were remarkable for a heart rate of 55 beats/minute and normal blood pressure, respiratory rate, and oxygen saturation. Physical examination revealed normal heart sounds and clear lungs. 

**Figure 1:**
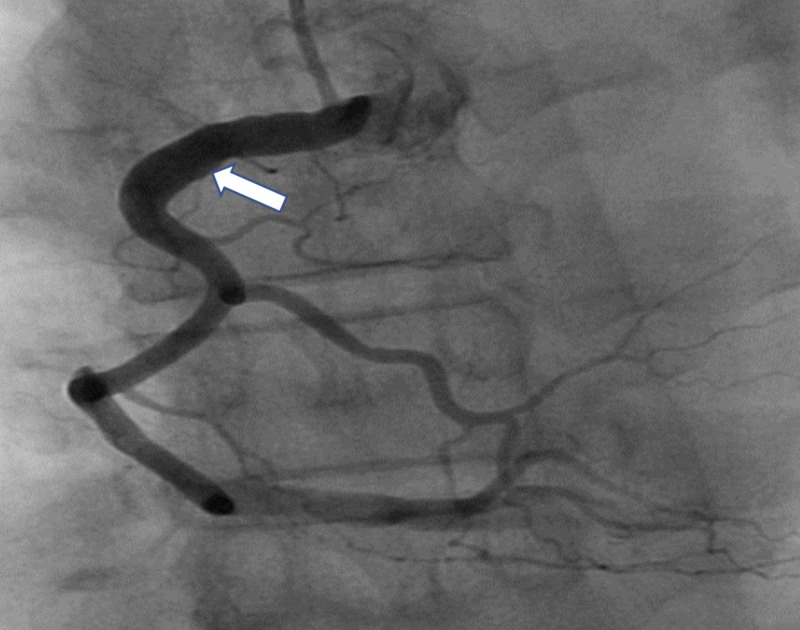
Plump and ectactic right coronary system

**Figure 2:**
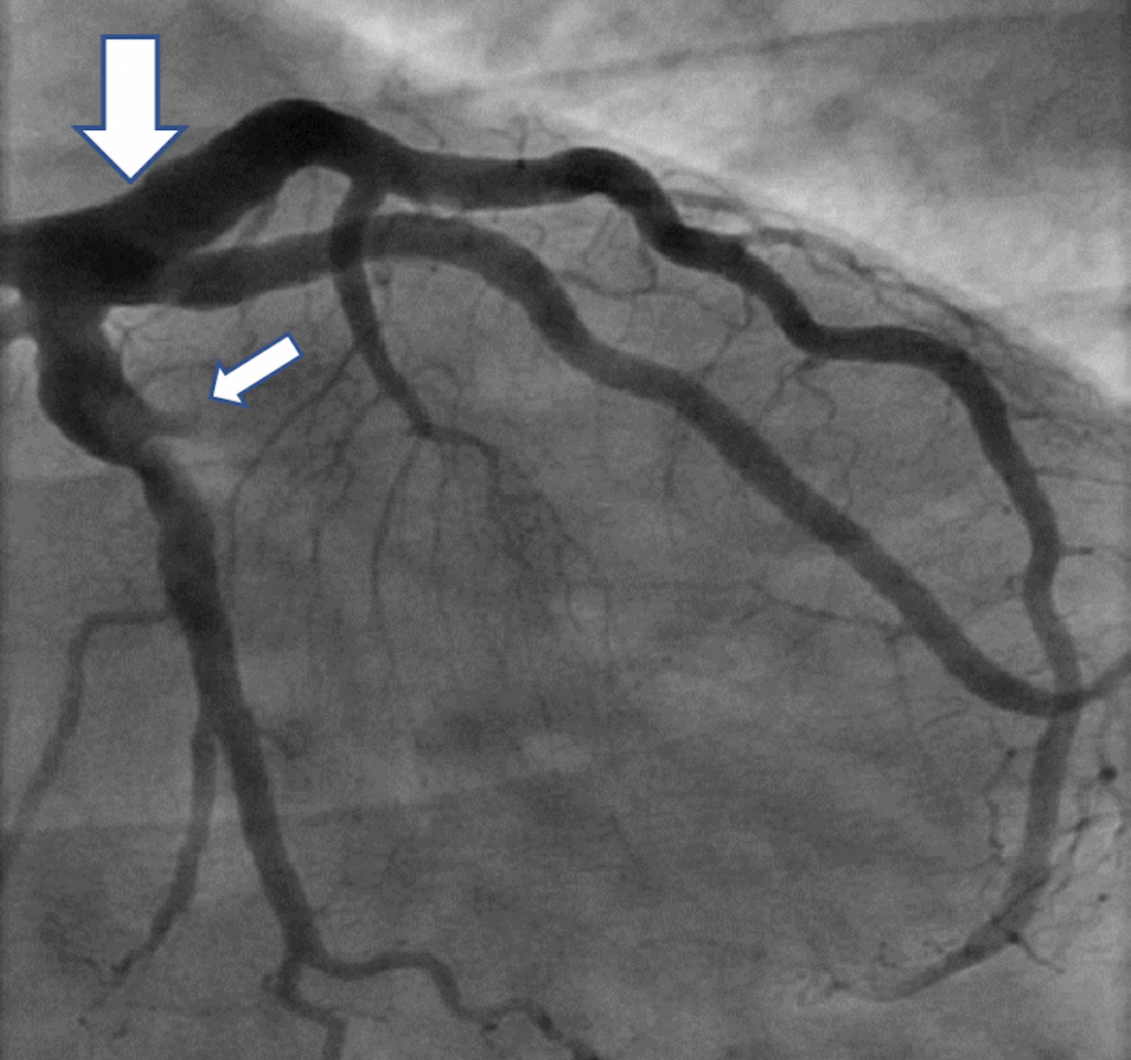
Ectactic left coronary system (large arrow) with thrombotic occlusion of the first obtuse marginal (OM-1) artery (small arrow)

**Figure 3:**
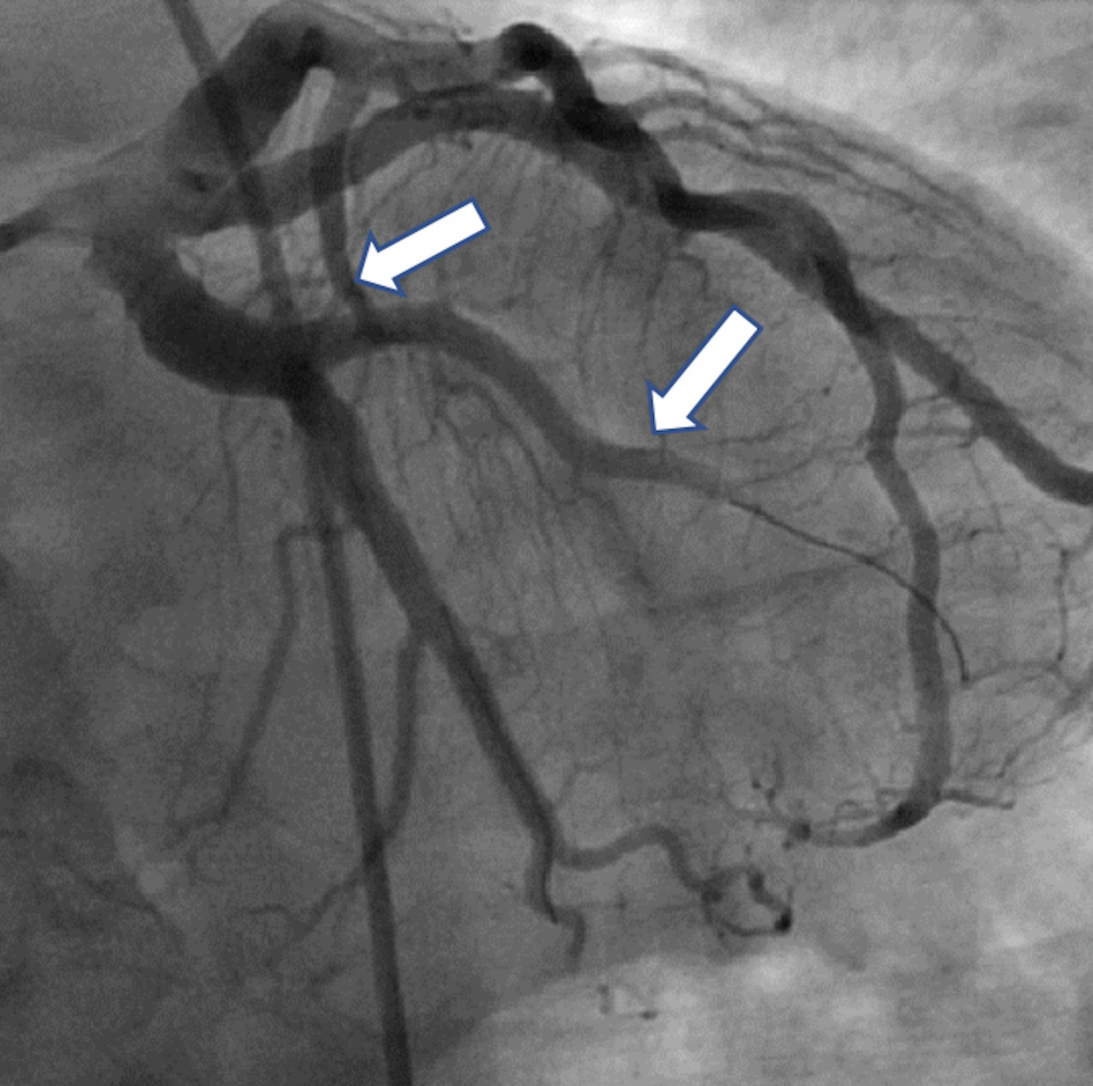
Balloon angioplasty and thrombectomy of the first obtuse marginal (OM-1) artery

**Figure 4:**
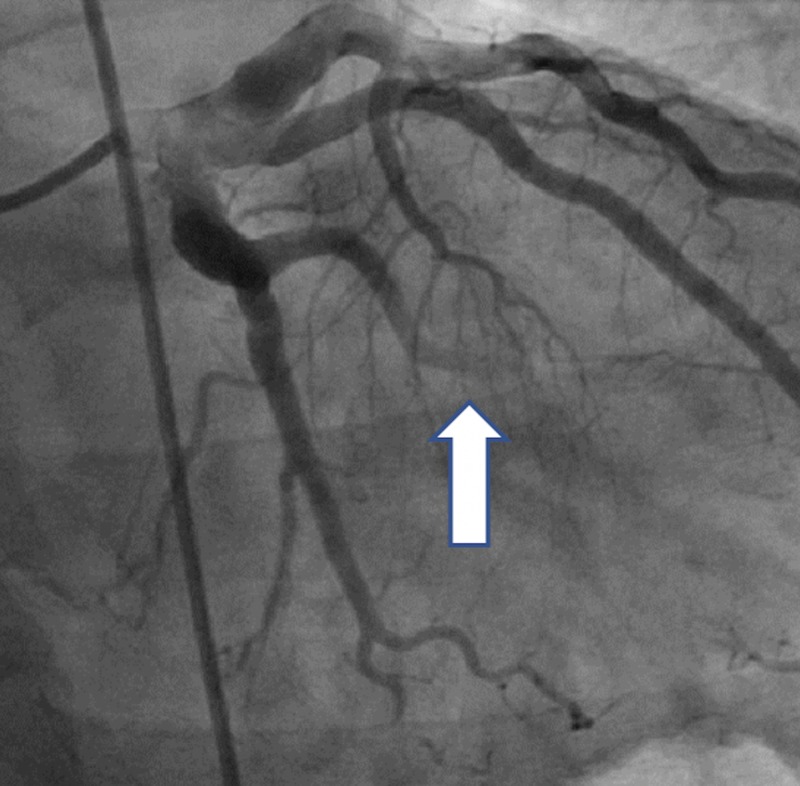
Post-thrombectomy distal TIMI flow = 1 TIMI: Thrombolysis in Myocardial Infarction

The initial electrocardiogram (ECG) showed sinus bradycardia with a first-degree atrioventricular (AV) block but without any ST-T wave changes. The initial troponin-T was negative and a total creatine kinase (CK) was 248. The patient received Plavix (600 mg), atorvastatin (80 mg), morphine for pain, and nitroglycerin and heparin infusions for presumed unstable angina. Beta-blocker was not given due to bradycardia. A subsequent ECG four hours later showed prominent Q-waves in the inferior leads and the troponin-T and CK rose to 0.2 and 624, respectively. Interventional Cardiology was consulted and the patient was taken to the catheterization lab for further management of the non-ST elevation myocardial infarction (NSTEMI). The coronary vessels on initial angiography were large and ectatic with visibly swirling blood flow (Figures 1-2). There was a 100% thrombotic occlusion of the first obtuse marginal (OM1) artery and a 60% thrombotic occlusion of the left circumflex artery (Figure 2). There was a 20% stenosis of the mid-left anterior descending (mid-LAD) artery and right coronary artery (RCA) as well. The culprit lesions in OM1 and circumflex arteries were treated with balloon angioplasty and with multiple rounds of manual thrombectomy yielding red thrombi (Figure 3). Interestingly, the post-intervention antegrade flow by Thrombolysis in Myocardial Infarction (TIMI) grade decreased in both vessels (TIMI 1), possibly due to the distal migration of the thrombi (Figure 4).

The patient received eptifibatide (180 mcg/kg double bolus) immediately before the initiation of PCI, followed by a continuous infusion of 2 mcg/kg/minute. The infusion was continued for 18 hours after which the patient was started on ticagrelor, 90 mg orally twice a day (maintenance dose), and continued on daily aspirin, high-intensity statin, a beta blocker, and Coumadin bridged with heparin. Echocardiography done on the following day showed basal lateral and basal-mid inferolateral wall akinesis and an estimated ejection fraction of 55.0%. Owing to a personal and family history of premature MI, the patient underwent extensive rheumatologic workup which included complement levels (C3 and C4), anti-myeloperoxidase antibody, anti-proteinase-3 antibody, anti-dsDNA-antibody, and anti-Smith antibody, but all results were unremarkable. Interestingly, the patient had an elevated antinuclear antibody (ANA) and a low positive Scl-70 antibody titer, but a final diagnosis of scleroderma or any other connective tissue disorder was not entertained given the absence of suggestive clinical signs and symptoms. The hypercoagulability workup was kept limited to JAK2 kinase mutation analysis, Factor-V Leiden, and prothrombin gene mutational analysis as the patient had received anticoagulants, as well as antithrombotics, in the acute setting. The patient also underwent MRA (magnetic resonance angiography) of the whole body which failed to show any aneurysmal dilation of vasculature elsewhere. The hospital course remained uneventful and the patient was discharged on aspirin, ticagrelor, and Coumadin after achieving therapeutic INR (international normalized ratio). During the one year follow-up period, the Coumadin was switched to rivaroxaban, ticagrelor was stopped after six months, and the patient was continued on guideline-directed medical therapy (GDMT) for coronary artery disease (CAD) with favorable outcomes. The patient has been playing full-court basketball games without any further complaints or hospitalization.

## Discussion

CAE is defined as the local or diffuse dilation of coronary arteries ≥ 1.5 times the diameter of the adjacent normal segment of the same vessel or the diameter of the patient’s largest coronary artery [1]. The prevalence of CAE by coronary angiography is estimated to be 0.3% to 4.9% [2]. CAE is more common in younger men and affects the right coronary artery more frequently than other coronary vessels [3]. In one study, significantly higher values of C-reactive protein (CRP) and carotid intimal thickness were found in patients with CAE with or without atherosclerosis when compared with normal coronaries or atherosclerosis without CAE. It was postulated that factors other than, or in addition to, atherosclerosis may play a role in the development of CAE [4]. In one study, a reduced level of Vitamin D was shown to be associated with CAE [5]. However, exaggerated inflammation, as seen in the form of either the human immunodeficiency virus (HIV) infection alone [6] or higher levels of uric acid (UA) and high sensitivity CRP (Hs-CRP) [7], elevated monocytes to high-density lipoprotein ratio (MHR) [8], a high neutrophil to lymphocyte (N/L) ratio [9], elevated insulin-like growth factor I (IGF-I) level [10], or high levels of inflammatory cytokines (i.e., IL-1b, tumor necrosis factor alpha (TNF-α), and interleukin 10 (IL-10) [11]), has been the main inciting factor in CAE development and progression. CAE often presents as exercise-induced angina; however, it is not uncommon to present as ACS as well [12-13]. The gold standard for diagnosis is coronary angiography where it is recognized by delayed anterograde contrast filling, local contrast deposition in artery segment (stasis), or segmental back-flow phenomenon [14]. Although computed tomography angiography (CTA) has been used for diagnosing this condition non-invasively, cardiac MRA has the advantage of avoiding radiation and iodinated contrast exposure and is proposed as a superior study for follow-up [15]. Yip et al. earlier reported the clinical features and outcome of CAE in patients with acute myocardial infarction (AMI) undergoing a primary PCI [16]. In their study, all of the infarct-related arteries (IRAs) were filled with heavy thrombi. The no-reflow phenomenon and distal embolization after primary PCI were found in 62.5% and 70.8% of IRAs, respectively. In addition, a large thrombus burden was also found to be associated with death, repeat AMI, re-intervention, and a higher risk of distal embolization and stent thrombosis. However, there have not been established guidelines either for removing the high thrombotic burden from ectactic IRAs during primary PCI or for secondary prevention. Thrombectomy with intracoronary thrombolysis can help remove most of the thrombus during primary PCI [17-18]. Conversely, the presence of a high thrombotic burden can pose a challenge for establishing the immediate blood flow owing to the thrombi migrating into distal coronary segments during thrombectomy attempts. In such instances, patients were put on extended duration oral anticoagulation and clot clearance was demonstrated with follow-up angiographies [19]. In one study, the occurrence rate of major adverse cardiac events (MACE) defined as cardiac death and nonfatal myocardial infarction during 49 months follow-up period after an acute MI was studied. The MACE rate of patients with CAE was significantly higher compared to those without CAE. Patients with CAE treated with oral anticoagulation (warfarin in this case) with the percentage of time in therapeutic range (%TTR) ≥ 60% demonstrated a lower occurrence of MACE compared with those with %TTR < 60% or without anticoagulation therapy [20].

The case we present had a high thrombus burden during PCI with a very low post-thrombectomy TIMI flow due to possible embolization of the thrombi into distal coronary segments. However, based upon the utility of longer duration oral anticoagulation (as described in the cited studies) in clearing the thrombi, as well as in reducing the occurrence of MACE, we opted to start our patient on extended duration oral anticoagulation rather than using intracoronary thrombolysis and multiple follow-up angiograms. The one-year follow-up results in our case were favorable. 

## Conclusions

Thus, it can be suggested to remove the maximum clot burden with mechanical thrombectomy alone and provide secondary prophylaxis with oral anticoagulation in addition to GDMT for CAD. Since there are no current guidelines regarding the best management of this disease, the future studies may address the duration as well as the choice of anticoagulation/antithrombin therapy.
